# Book Reviews, Pamphlets, Exchanges

**Published:** 1901-09

**Authors:** 


### *BOOK REVIEWS, PAMPHLETS, EXCHANGES.*

---

The Ready-Reference Hand-Book of Skin Diseases. By George Thomas Jackson, M.D., Chief of Clinic and Instructor in Dermatology, College of Physicians and Surgeons, New York. New (4th) edition, thoroughly revised. In one 12mo. volume of 617 pages, with 82 engravings and 3 colored plates. Cloth, $2.75, net. Lea Brothers & Co., Philadelphia and New York, 1901.

The necessity for a fourth edition of this work within a short time of the appearance of the third is evidence of its popularity. The illustrations are very good, save the diagrams of skin, primary and secondary, lesions.

The sections on therapeutics is very complete and practical though brief. The entire text is necessarily brief, owing to the size of the work. Descriptions of some new and mostly rare diseases are added in this edition.

The alphabetical arrangement of subjects is continued. In spite of the many criticisms of this plan it is best for a work of the kind, if for no other reason than the avoidance of the confusion of a confounded classification. The book should be owned by every practitioner who is interested in the subject.

---

Uterine Fibromata, their Pathology, Diagnosis and Treatment. By E. Stanmore Bishop, F.R.CDS. England, Philadelphia. P. Blakiston's Son & Co. 1901.

This book undertakes to present a comprehensive view of the literature of uterine fibromata. The progress in special surgery has rendered a work of this character necessary, as the facts connected with the subject are collected together and readily accessible. The book is well illustrated, containing a number of very clear drawings, showing the gross changes in the pathology of the disease and elucidating the surgical technique. Surgeons and gynecalogists will find in this treatise much valuable information, and the general practitioner who is concerned in concervative gynecology will also find profit in reading the book.

---

CLINICAL PATHOLOGY OF THE BLOOD. By James Ewing, M.D., A.M., Professor of Pathology in Cornell University Medical College. Octavo, 432 pages. Lea Bros. & Co., Philadelphia and New York.

This book is very opportune, as it occurs at a time when so much interest is being displayed in a practical way in the morphology and pathology of the blood. The book forms a very complete study. It is well illustrated, containing many colored plates and will doubtless prove a valuable addition to the literature of the subject. It can be recommended as a good guide for the kind of work concerned.

---

### ULCERATED CORNEA.

A most satisfactory treatment of corneal ulcerations is by means of the actual cautery. This may be used in the form of the electrocautery, thermocautery, or by an instrument heated in an alcohol lamp. As a rule I prefer the latter method. Any small, smooth piece of steel, large silver probe, or knitting-needle is much easier handled by the novice than the clumsy cautery handles with their stiff, attached conducting cords. In the use of these cauteries great care must be used not to puncture the cornea, and where the ulcer is deep, this is a delicate operation. By means of a good light and with a delicate touch, we can usually avoid this accident. It has only happened to me once in my seventeen years of eye practice. Persons without a steady hand and delicate touch had better not attempt it, or, for that matter, not much other eye surgery.

After cautery atropine should be used. As a rule, one cauterization is sufficient, but at times there occur cases where, in spite of repeated cauterization, and all other means, the disease goes on to perforation or to complete destruction.—*Northwestern Lancet.*

